# Migalastat Tissue Distribution: Extrapolation From Mice to Humans Using Pharmacokinetic Modeling and Comparison With Agalsidase Beta Tissue Distribution in Mice

**DOI:** 10.1002/cpdd.941

**Published:** 2021-04-19

**Authors:** Yi Shuan Wu, Richie Khanna, Virginia Schmith, Yi Lun, Jin‐Song Shen, Anadina Garcia, Leo Dungan, Anthony Perry, Lukas Martin, Pai‐Chi Tsai, Rick Hamler, Anibh M. Das, Raphael Schiffmann, Franklin K. Johnson

**Affiliations:** ^1^ Nuventra Pharma Sciences Durham North Carolina USA; ^2^ Amicus Therapeutics, Inc. Cranbury New Jersey USA; ^3^ Clinic for Paediatric Nephrology Hepatology and Metabolic Disorders Hannover Medical School Hannover Germany; ^4^ Baylor Scott and White Research Institute Dallas Texas USA

**Keywords:** agalsidase beta, biodistribution, Fabry disease, migalastat, pharmacokinetics, pharmacological chaperone, physiologically based pharmacokinetic modeling

## Abstract

Approved therapies for Fabry disease (FD) include migalastat, an oral pharmacological chaperone, and agalsidase beta and agalsidase alfa, 2 forms of enzyme replacement therapy. Broad tissue distribution may be beneficial for clinical efficacy in FD, which has severe manifestations in multiple organs. Here, migalastat and agalsidase beta biodistribution were assessed in mice and modeled using physiologically based pharmacokinetic (PBPK) analysis, and migalastat biodistribution was subsequently extrapolated to humans. In mice, migalastat concentration was highest in kidneys and the small intestine, 2 FD‐relevant organs. Agalsidase beta was predominantly sequestered in the liver and spleen (organs unaffected in FD). PBPK modeling predicted that migalastat 123 mg every other day resulted in concentrations exceeding the in vitro half‐maximal effective concentration in kidneys, small intestine, skin, heart, and liver in human subjects. However, extrapolation of mouse agalsidase beta concentrations to humans was unsuccessful. In conclusion, migalastat may distribute to tissues that are inaccessible to intravenous agalsidase beta in mice, and extrapolation of mouse migalastat concentrations to humans showed adequate tissue penetration, particularly in FD‐relevant organs.

Fabry disease (FD) is a rare lysosomal storage disorder caused by any of several hundred variants of the X‐chromosomal *GLA* gene, which results in functional deficiency of the lysosomal enzyme α‐galactosidase A (α‐Gal A) and progressive accumulation of glycosphingolipids, particularly globotriaosylceramide (GL‐3), in diverse cell types throughout the body.^1^ Supraphysiologic accumulation of glycosphingolipids triggers a positive feedback of chronic inflammation and cellular damage with serious clinical sequelae in many organ systems,^2^ including but not limited to neurological, cardiac, ocular, gastrointestinal, dermatological, auditory, renal, and cerebrovascular organ systems, with premature death in many patients.^1^, ^3^

Because of the multisystemic nature of FD, the ideal therapeutic agent must achieve broad tissue distribution and adequate tissue penetration. Treatments for FD include intravenous enzyme replacement therapy (ERT) with agalsidase beta or agalsidase alfa and the oral pharmacological chaperone migalastat.^4^ ERT exogenously replaces the deficient lysosomal enzyme and has been a mainstay of treatment for nearly 20 years. Exogenous agalsidase is expected to be taken up into the lysosomes of most tissues by receptor‐mediated endocytosis via cation‐independent mannose‐6‐phosphate receptors (M6PRs) on the extracellular membrane,^5^, ^6^ whereas hepatic and splenic uptake of agalsidase is thought to be driven by macrophage mannose receptors on the plasma membrane.^5^ Similar to other protein biologics, agalsidase is expected to be eliminated by peptide hydrolysis. However, evidence suggests that agalsidase alfa and agalsidase beta may only superficially penetrate the endothelial cells of various target organs. A nonclinical study of agalsidase alfa in a mouse model of FD showed distribution in liver, spleen, adrenal glands, kidneys, heart, testes, and bone marrow, but not in other relevant tissues such as skin, intestines, eyes, or brain.^7^ Tissue biopsies from patients with FD receiving long‐term agalsidase beta treatment showed some GL‐3 reduction in kidney, heart endothelial cells, and skin, but GL‐3 levels remained high in noncapillary smooth muscle cells, podocytes of the kidney, and cardiomyocytes.^8^, ^9^, ^10^ In addition, both forms of ERT are predominantly distributed to the liver and then to the spleen,^11^ neither of which is a key organ in Fabry pathogenesis, prompting research to improve agalsidase biodistribution.^12^, ^13^, ^14^, ^15^

Migalastat is a small molecule that stabilizes *amenable* endogenous mutant forms of α‐Gal A for trafficking to the lysosomes, on which migalastat dissociates, and allows the enzyme to metabolize the glycosphingolipids.^16^ In human studies, migalastat has absolute oral bioavailability of 75%, exhibits dose‐proportionality between 75 and 1250 mg, and is primarily renally eliminated unchanged with minor metabolism by uridine diphosphate glucuronosyltransferase. In in vitro experiments, migalastat has no detectable protein binding in human plasma and is not a substrate of any of the major transporters. In mouse models, migalastat reduced GL‐3 concentrations in kidneys, heart, brain, skin, and plasma.^17^, ^18^ Nonclinical findings were consistent with the results of phase 3 studies, which showed that patients with FD treated with migalastat had partial clearance of GL‐3 in renal kidney interstitial capillaries and podocytes,^19^, ^20^ stabilized renal function, significant reduction in cardiac mass as measured by left ventricular mass index (LVMi),^21^, ^22^ and improvement in gastrointestinal symptoms.^19^

The efficacy of migalastat depends on adequate penetration to various target tissues. Although the pharmacokinetics of migalastat in plasma have been thoroughly assessed in healthy subjects and patients with FD,^16^, ^23^, ^24^, ^25^, ^26^ migalastat concentrations in disease‐relevant tissues and a comparison of these concentrations with those reached after administration of ERT cannot be investigated in humans but can be assessed in animals. Physiologically based pharmacokinetics (PBPK) modeling is a mechanistic modeling approach that allows for the extrapolation of animal data into human predictions. PBPK uses anatomical and physiological information of an organism and physicochemical and biological knowledge about a drug to predict drug disposition in all organ and tissue compartments. The information on the organism can be substituted to allow for extrapolation of the PBPK model to other populations.

The use of PBPK models developed in animals has aided in tissue distribution studies of various drugs.^27^, ^28^, ^29^ For example, a PBPK model initially developed in mice was extrapolated to humans to successfully predict tissue concentrations of an anticancer agent and estimate its efficacy.^27^ Similarly, PBPK modeling in rats has been instrumental for predicting antibiotic penetration in several human tissues and assessing the need for adverse drug reaction monitoring.^28^, ^29^ By enabling predictions of drug availability in target organs, PBPK models may provide valuable insights regarding drug availability in target tissues that may correlate with efficacy and safety.

The present report describes nonclinical pharmacokinetic studies evaluating target tissue concentrations of migalastat and agalsidase beta over time in mice, their characterization using PBPK modeling, and comparison of tissue concentrations over time in mice between agents. In addition, the extrapolation of tissue concentrations from mice to humans was attempted to predict human tissue concentration‐time data to provide insight into our understanding of efficacy in FD‐relevant organs, if possible.

## Methods

### Materials

The hR301Q α‐Gal A transgenic (Tg)‐knockout (KO) mice were obtained from Dr. Robert Desnick (Mount Sinai School of Medicine, New York, New York). Wild‐type C57BL/6 mice were purchased from Taconic Farms (Germantown, New York). Animal husbandry and all experiments were conducted under Institutional Animal Care and Use Committee‐approved protocols. The hydrochloride salt of migalastat was synthesized by Cambridge Major Laboratories (Germantown, Wisconsin). Agalsidase beta was chosen based on its availability in the United States and Europe and was purchased from Sanofi Genzyme (Cambridge, Massachusetts). All other reagents were purchased from Sigma Aldrich (St. Louis, Missouri), unless noted otherwise.

### Migalastat and Agalsidase Beta Experiments in Mice

A total of 102 male 8‐week‐old C57BL/6 mice (mean weight, 25 g) were divided into 4 cohorts and were administered a single dose of 3, 10, 30, or 100 mg/kg migalastat. At each sample collection time, 3 mice were euthanized by carbon dioxide prior to sample collection. The first cohort consisted of 30 mice, which were intravenously administered 3 mg/kg migalastat (5 mL/kg of 0.6 mg/mL migalastat in saline), and plasma sampling was conducted 0.083, 0.25, 0.5, 0.75, 1, 2, 3, 4, 8, and 24 hours after the dose. Cohorts 2‐4 each included 24 mice that received 10, 30, or 100 mg/kg migalastat (diluted in 10 mL/kg of water) by oral gavage, and plasma sampling was conducted 0.25, 0.5, 1, 2, 3, 4, 8, and 24 hours after dose. For cohort 4, brain, heart, kidney, and skin concentrations were also assayed at each timepoint.

For pharmacokinetic assessment at steady state, 2 cohorts of 48 male C57BL/6 mice each, 12 weeks old at study initiation, were administered either 30 mg/kg migalastat orally on Monday, Wednesday, and Friday for 12 doses or 1 mg/kg agalsidase beta by intravenous tail injection every other week for 2 doses. At 1, 2, 4, 6, 8, 24, 48, and 72 hours following the last dose, 6 mice were euthanized with carbon dioxide, and plasma, brain, heart, kidney, liver, small intestine, and spleen tissue samples were collected.

Biodistribution of migalastat and agalsidase beta was also assessed 2 hours after dose in a mouse model of FD (10‐ to 12‐week‐old hR301Q α‐Gal A Tg‐KO mice) for comparison of the migalastat and agalsidase beta concentrations with those of wild‐type mice. Two cohorts of 5 hR301Q α‐Gal A Tg‐KO mice each received the same migalastat or agalsidase beta treatment as described for the steady‐state experiment. Two hours after dose, these mice were euthanized with carbon dioxide, and plasma, brain, heart, kidney, liver, small intestine, and spleen tissue samples were collected.

### Sample Collection

On sacrifice of the mice, whole blood was drawn into lithium heparin tubes from the inferior vena cava, then immediately centrifuged at 2700*g* for 10 minutes at 4°C to collect plasma. Mice were then necropsied and shaved, and tissues including brain, heart, kidneys, liver, skin, small intestine, and spleen were quickly removed from an incision made from the lower ventral neck to the abdominal cavity. Tissues were rinsed in cold phosphate‐buffered saline to wash off any excess blood, blotted dry, and stored on dry ice for assessment of migalastat and agalsidase beta levels.

### Quantitation of Migalastat Levels

To quantitate migalastat levels, mouse tissues were homogenized and extracted using 300 μL of water per 100 mg of all tissues (except skin, for which 500 μL of 1:1 water: methanol per 100 mg tissue was used) and a FastPrep homogenizer (MP Biomedical, Irvine, California) followed by centrifugation at 10 600*g* for 5 minutes at 4°C. An equal volume of acetonitrile (ACN):water (95:5) was added to 25 μL of tissue homogenate and then spiked with 50 ng/mL d2‐migalastat ^13^C‐hydrochloride internal standard (MDS Pharma Services, Lincoln, Nebraska). Each sample was vortexed and centrifuged at 10 000*g* for 5 minutes at room temperature. From each sample, migalastat level was determined from 15 μL of supernatant. No homogenization was conducted for plasma samples, and an equal volume of ACN:water (95:5) was added to 25 μL of plasma samples for extraction as described above for migalastat assay.

Migalastat concentrations were assayed using liquid chromatography (LC; Tosoh Bioscience, Cincinnati, Ohio)–tandem mass spectroscopy (MS/MS; Sciex API 3000 MS/MS; AME BioSciences, Toten, Norway), as described previously with slight modifications.^17^ Briefly, LC was conducted using an ACN:water:formate binary mobile phase system (mobile phase A: 5 mm ammonium formate, 0.05% formic acid in 95:5 ACN:water; mobile phase B: 5 mm ammonium formate, 0.05% formic acid in 55:45 ACN:water) with a flow rate of 0.6 mL/min on an amide‐80 column (50 × 2 mm, 5 μm; Tosoh Bioscience, Cincinnati, Ohio). The MS/MS analysis was carried out under atmospheric‐pressure chemical ionization positive ion mode. The following transitions were monitored: mass/charge (m/z) 164.2→80.10 for migalastat and m/z 167.2→80.10 for the internal standard. An 11‐point calibration curve and quality control samples were prepared in the same manner as the samples. The ratio of the area under the curve (AUC) for migalastat to that of the internal standard was determined, and final concentrations of migalastat in each sample were calculated using the linear least‐squares fit equation applied to the calibration curve. To derive approximate molar concentrations, 1 g of tissue was estimated as 1 mL of volume. The in‐process stability of samples was ≤24 hours. Long‐term stability of frozen plasma and tissue samples stored at −80°C was >1 year. Range of quantification was 0.5 to 1000 ng/mL for plasma and kidney tissue and 1.0 to 1000 ng/mL for heart, brain, spleen, small intestine, and liver. Precision and accuracy were within 20%.

### Quantitation of Agalsidase Beta Levels

Agalsidase beta levels were quantitated as a measure of α‐Gal A activity, using previously described methods with slight modifications.^17^, ^26^ Briefly, tissue lysate was prepared by homogenization of ∼50 mg tissue for 3‐5 seconds on ice with a microhomogenizer (Pro Scientific, Thorofare, New Jersey) in 200 μL of lysis buffer (0.1% Triton X‐100, 27 mM sodium citrate, 46 mM sodium phosphate dibasic, pH 4.6). Lysate (20 μL) was added to 50 μL of assay buffer (27 mM sodium citrate, 46 mM sodium phosphate dibasic, 12 mM 4‐methylumbeliferryl‐α‐d‐galactopyranoside (4‐MUG), 90 mM N‐acetyl‐d‐galactosamine, pH 4.6) and incubated for 3 hours at 37°C. Reactions were stopped by the addition of 70 μL 0.4 M glycine, pH 10.8, and fluorescence was read at 460 nm on a Victor^3^ plate reader (Perkin Elmer, Waltham, Massachusetts) after excitation at 355 nm. Raw fluorescence counts were background‐subtracted (defined by assay buffer only).

A bicinchoninic acid protein assay (Pierce, Rockford, Illinois) was used to determine total protein concentration in tissue lysates.^30^ A 4‐methylumbelliferone (4‐MU) standard curve ranging from 50 mM to 30 mM was run each day for conversion of fluorescence counts to absolute α‐Gal A activity, expressed as nanomoles of released 4‐MU per milligram of total protein per hour (nmol/mg protein/h). Activity units were converted into concentration (ng/mL) using specific activity of agalsidase beta that was calculated using the 4‐MUG assay as described above. The in‐process stability of samples after the addition of stop buffer was ≤2 hours. Long‐term stability of frozen plasma and tissue samples stored at −80°C was >1 year. Precision and accuracy were within 20%.

### Pharmacokinetic Study in Humans

The distribution of migalastat and agalsidase beta in human tissues has not been studied, but plasma migalastat and agalsidase beta data are available to allow extrapolation of tissue concentrations from mice to humans with observed data in humans. Plasma migalastat concentration‐time profiles in healthy human subjects and patients with FD were available from a phase 1 study (AT1001‐015; NCT01730469)^16^ and the phase 3 FACETS study (AT1001‐011; NCT00925301),^19^ respectively. Agalsidase beta concentration‐time profiles in patients with FD were assessed in a phase 2a study (NCT01196871).^26^

### PBPK Modeling of Migalastat Concentration‐Time Data in Mice

A small‐molecule PBPK model of migalastat in mice was built using the standard whole‐body 15‐organ model implemented in PK‐Sim (version 8 build 22; Bayer, Leverkusen, Germany) of the Open Systems Pharmacology Suite.^31^ A mouse weight of 25 g was assumed for simulation. Physicochemical properties were obtained from in vitro data and iteratively optimized based on observed concentrations as appropriate. Given that migalastat was not a substrate of any important transporters except sodium‐glucose cotransporter 1, active renal processes were assumed to be negligible, and migalastat was assumed to be freely filtered at glomeruli, with the rest of the clearance accounted for by hepatic metabolism. A protein‐binding partner was added to account for apparent tissue retention compared with plasma. Although brain concentrations were low, the predicted brain concentrations were not eliminated; therefore, a minor glucuronidation process mediated by low concentration of brain uridine 5’‐diphosphate‐glucuronosyltransferase (UGT) was incorporated to account for a slow elimination from the brain. Model parameters were adjusted until plots of observed and predicted concentrations in each important tissue showed reasonable agreement.

Population simulations were conducted in mice by first simulating 1000 individuals with variability in demographic attributes and biological processes. Mouse weight was allowed to vary from 20 to 30 g, with 30% coefficient of variation (CV) allowed on the tissue lysosome level, brain UGT concentration, renal function, hepatic metabolism, and specific intestinal permeability and 15% CV allowed on the partition coefficients of brain, heart, kidney, liver, muscle, skin, and small intestine to account for uncertainty and variability in volume of distribution. Then, mouse exposure was simulated assuming doses of 100 or 30 mg/kg every other day.

### Prediction of Human Tissue Concentration‐Time Data Through Extrapolation of the Migalastat PBPK Model From Mice to Humans

**Figure 1:**
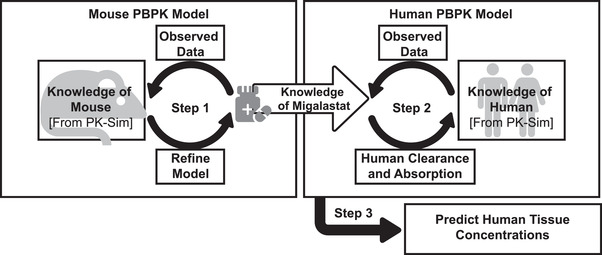
Development of physiologically based pharmacokinetic (PBPK) models to extrapolate mouse migalastat concentrations to humans. The mouse PBPK model for migalastat was generated and refined using observed mouse plasma and tissue concentration‐time data. PK properties of migalastat from this model informed development of the human PBPK model, which was further refined with observed human plasma and tissue migalastat concentration‐time data to predict migalastat concentrations in human tissues.

Each model was extrapolated to a PK‐Sim default of a 73‐kg European male by optimizing absorption and clearance to human plasma data. This new human model with adjusted absorption and clearance was used to predict tissue concentrations (Figure 1).

Population simulations were conducted in humans by first simulating 1000 individuals with variability in demographic attributes and biological processes. The simulation assumed an equal number of males and females, aged 20‐60 years, with weight based on age‐ and sex‐based weight distribution in PK‐Sim, 45% CV on the biological processes, and 30% CV on the partition coefficients. The 15% higher CV in humans compared with mice was incorporated to account for uncertainty in the mouse‐to‐human extrapolation and the increased variability in humans compared with mice. Human exposures in plasma and tissue were simulated assuming a dose of 123 mg migalastat every other day. The predicted human plasma concentration‐time curve was compared with the observed plasma concentration‐time curve data from study AT1001‐015^16^ and the phase 3 FACETS study.^19^

### PBPK Modeling of Agalsidase Beta Data in Mice

Baseline α‐Gal A activity in each tissue was subtracted from the total α‐Gal A activity at each timepoint to determine the concentrations attributed to exogenous agalsidase beta administration prior to conducting PBPK modeling. Agalsidase beta was modeled as a protein or large molecule in MoBi (Bayer, Leverkusen, Germany). Receptor‐mediated tissue uptake was incorporated by adding a receptor on the extracellular membrane; then a first‐order process was implemented to allow for conversion of interstitial complexes of agalsidase beta and receptor into free agalsidase beta within the cell. In addition, small metabolism processes were implemented in interstitial and intracellular compartments to account for the ubiquitous peptide hydrolysis. A mouse weight of 25 g was assumed for conducting the simulation.

### Prediction of Human Tissue Concentration‐Time Data Through Extrapolation of the Agalsidase Beta PBPK Model From Mice to Humans

Human extrapolation was performed assuming a 30‐year‐old 73‐kg European man. Because agalsidase beta was intravenously administered, no parameters were further optimized in the mouse‐to‐human extrapolation. The predicted human plasma agalsidase beta concentration‐time data were compared with observed data in patients with FD in a phase 2a study (NCT01196871).^26^

## Results

### Migalastat and Agalsidase Beta Biodistribution in Mouse Tissue

**Figure 2:**
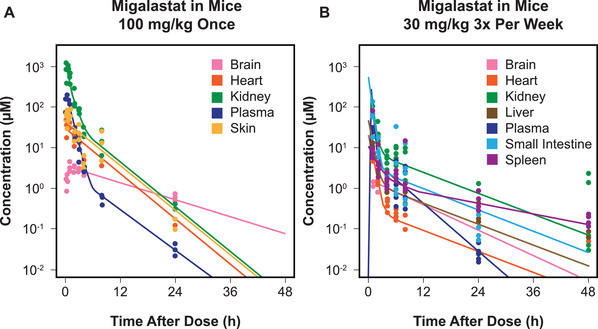
Migalastat pharmacokinetics in wild‐type mice following (A) single and (B) steady‐state dosing. h, hour. Migalastat concentrations were assessed in wild‐type mice following a single oral dose (100 mg/kg) or steady‐state dosing (30 mg/kg, 3 times per week for 12 doses) and were measured 0.083, 0.25, 0.5, 0.75, 1, 2, 3, 4, 8, and 24 hours postdose (data from 3‐6 mice for each value). Lines were fitted to the data based on biexponential decline, with rounding of the terminal elimination kinetic constant to reduce noise.

Following single‐dose or steady‐state migalastat administration in wild‐type mice, plasma concentrations of migalastat declined more rapidly than tissue concentrations (Figure 2). Migalastat showed broad distribution, particularly in FD‐relevant tissue. Kidneys had the highest concentrations, followed by small intestine, spleen, and liver, with similar concentrations in skin and heart and low concentrations in the brain. Two hours after dose, migalastat concentrations were comparable between wild‐type and hR301Q α‐Gal A Tg‐KO mice in the brain, heart, and spleen, but the mutant mice had lower concentrations in the kidneys, liver, and small intestine (Figure S1).

**Figure 3:**
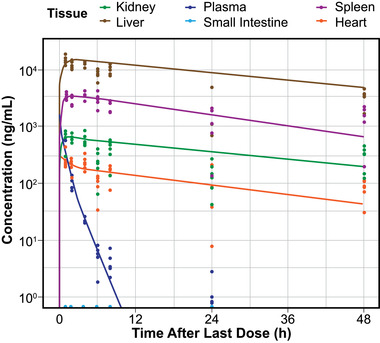
Observed and physiologically based pharmacokinetic (PBPK) model‐predicted agalsidase beta concentrations in mice following intravenous administration of 1 mg/kg agalsidase beta every other week. h, hour. Dots are observed values; lines are PBPK model predictions. A mouse PBPK model for agalsidase beta was developed and fitted to observed mouse agalsidase beta concentrations following steady‐state intravenous dosing (1 mg/kg biweekly) for 2 doses. Agalsidase beta concentration in the brain was below the limit of detection.

Agalsidase beta demonstrated a typical PK profile for mannose‐6‐phosphate phosphorylated lysosomal enzymes (Figure 3; observed data). Plasma concentrations fell rapidly after drug administration because of rapid distribution of most of the administered dose into the liver and spleen, with lower concentrations in the disease‐relevant tissues of the kidney and heart and undetectable levels in the small intestine and brain. Tissue levels of agalsidase beta decreased slowly for the duration of assessment (72 hours). Agalsidase beta concentrations were compared in tissues of wild‐type and hR301Q α‐Gal A Tg/KO mice 2 hours postdose, and concentrations were comparable in the liver, spleen, kidney, and heart (Figure S2).

### PBPK Model Fit of Migalastat Compared With Observed Data in Mice

During model development, it became clear that the model using the full dataset resulted in a better fit for the 10‐, 30‐, and 100‐mg/kg single‐dose data resulted, whereas the model using steady‐state data alone resulted in a better fit for the 30 mg/kg steady‐state data (Figure S3). These differences could not be explained by nonlinearity in elimination, as a lower dose was used for the steady‐state study, and the oral 30 mg/kg single‐dose data had an elimination half‐life similar to the other oral single‐dose data but was eliminated more quickly than the 30 mg/kg steady‐state data. The single‐dose studies showed that migalastat exhibits dose‐proportionality between oral 10 and 100 mg/kg in mice. The steady‐state study had an average of 6 mice per timepoint, whereas the single‐dose data had an average of 3 mice per timepoint but included 4 doses (intravenous 3 mg/kg, oral 10 mg/kg, oral 30 mg/kg, and oral 100 mg/kg). The steady‐state data were assessed over a longer time after dose, which may allow better interpretation of the terminal elimination of migalastat. Both models overpredicted exposures for the intravenous data. The apparently lower bioavailability of the intravenous data was contrary to expectation; however, the intravenous data were based on a lower dose, and distribution may still be taking place at the last quantifiable concentration. The decision was made to develop models based on either the full dataset (Full Dataset PBPK Model) or steady‐state data alone (Steady‐state Dataset PBPK Model) to provide a range of predictions. The important parameters in the 2 migalastat PBPK models are summarized in Table 1.

**Table 1:** Parameters for the Migalastat Physiologically Based Pharmacokinetic (PBPK) Model

Parameter	Initial Value	Source/Rationale	Full Dataset Model	Steady‐State Dataset Model
**Basic physicochemical properties**				
Is a small molecule	Yes	Not a protein or biologics drug	Fixed	Fixed
Lipophilicity^a^	−0.80	Fitted	−1.70	‐3.03
Binds to	None	Sponsor data on file	Fixed	Fixed
Fraction unbound	1.0	Sponsor data on file	Fixed	Fixed
Molecular weight, g/mol	163.17	Molecular structure of migalastat base	Fixed	Fixed
Has halogens	No	Molecular structure of migalastat base	Fixed	Fixed
Compound type	Monoprotic base	Sponsor data on file	Fixed	Fixed
PKa, basic	7.47	Sponsor data on file	Fixed	Fixed
Solubility	500 g/L at pH 1.2 to 7.5	Sponsor data on file	Fixed	Fixed
Partition coefficient	Rodgers & Rowland	Fitted	Schmitt	Schmitt
**Mouse‐specific biological properties**				
Specific intestinal permeability, cm/min	Program prediction^b^	Fitted	5 × 10^−4^	5 × 10^−4^
Specific binding–lysosome, K_d_ (mM)	0.001	Assumed	10	10
Specific binding–lysosome, K_off_ (1/min)	1.0	Fitted	1.6 × 10^−3^	2 × 10^−3^
Renal clearance	GFR = 100%	Assumed	Fixed	Fixed
Hepatic clearance–specific clearance, min^‐1^	0	Fitted	0.09	0.02
Extrahepatic clearance–UGT in brain CL_spec_/[enzyme], (L/μmoL/min)	0	Fitted	2.10 × 10^−3^	2.10 × 10^−3^
**Tissue partition coefficient, intracellular:plasma**				
Heart	Program prediction	Fitted	0.01	5 × 10^−3^
Kidney	Program prediction	Fitted	4.00	0.76
Liver	Program prediction	Fitted	0.10	5 × 10^−3^
Muscle	Program prediction	Fitted	0.50	0.79
Skin	Program prediction	Fixed	0.54	0.54
Brain	Program prediction	Fixed	0.81	0.80
**Human‐specific biological properties**				
Specific intestinal permeability, (cm/min)	Program prediction^b^	Fitted	1 × 10^−4^	2 × 10^−5^
Renal clearance	GFR = 100%	Assumed	Fixed	Fixed
Hepatic clearance–specific clearance, (min^‐1^)	0	Fitted	0.05	0.04

CL, clearance; GFR, glomerular filtration rate; K_d_, equilibrium dissociation constant; K_off_, off‐rate constant; min, minute; pKa, negative logarithmic of the acid dissociation constant; UGT, uridine 5′‐disphospho‐glucuronosyltransferase.

^a^ Lipophilicity is a highly uncertain parameter; ideal lipophilicity measure is membrane affinity but was not available; several log‐magnitude differences based on alternate lipophilicity measure may be possible.

^b^ PK‐Sim algorithm for predicting specific intestinal permeability is often significantly underestimated.

Based on the physicochemical attributes of migalastat as a small molecule, migalastat is expected to freely diffuse across cell membranes; therefore, plasma and tissue concentrations are expected to fall in parallel during the terminal phase. However, migalastat tissue concentrations were eliminated more slowly than plasma concentrations, with the tissue concentrations being roughly parallel to each other except for the spleen, which had even slower elimination. This phenomenon was modeled by adding tissue binding because of lysosomal sequestration. Tissue binding in spleen was not modeled, given that the spleen was not a highly disease‐relevant organ for FD.

**Figure 4:**
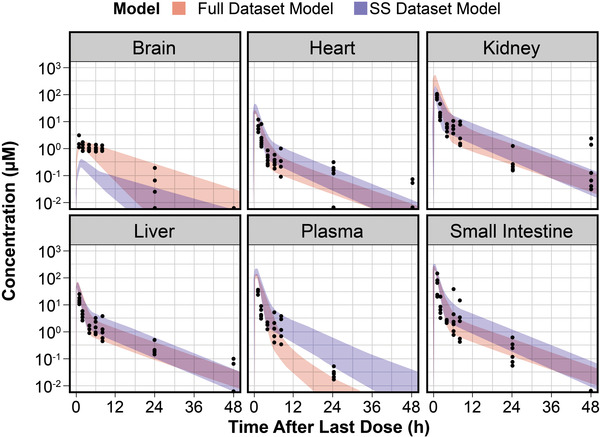
Observed and physiologically based pharmacokinetic (PBPK) model‐predicted migalastat tissue and plasma concentration‐time profiles in mice following steady‐state administration of 30 mg/kg oral migalastat 3 times per week. h, hour; SS, steady‐state. Dots are observed values; Color strips indicates 95% prediction interval. A mouse PBPK model was developed and fitted to observed mouse migalastat concentrations following steady‐state oral dosing (30 mg/kg 3 times per week for 12 doses).

The Full Dataset PBPK Model reasonably predicted all migalastat tissue concentrations for both the single‐dose (Figure S4) and steady‐state data (with the exception that plasma elimination was predicted to be faster than observed data), whereas the Steady‐state Dataset PBPK Model underpredicted brain concentrations but more accurately predicted plasma, kidney, and heart concentrations at steady state (Figure 4). Together, these 2 models predicted the range of possible concentration‐time data in each tissue of interest.

In the heart, kidney, liver, and small intestine, migalastat concentrations were summed from unbound concentrations and tissue‐bound concentrations believed to be sequestered in lysosomes. The unbound concentrations were proportional to the plasma concentration by a tissue‐specific constant (partition coefficient) throughout time. The tissue‐bound concentrations both increased and declined more slowly than unbound concentrations with a half‐life of 6.5 hours. For each tissue, the peak concentration predominantly consisted of unbound migalastat, whereas terminal elimination‐phase concentrations predominantly consisted of tissue‐bound migalastat (Figure S4).

### Predicted Migalastat Tissue Concentrations in Humans Using Mouse‐to‐Human Extrapolation

**Figure 5:**
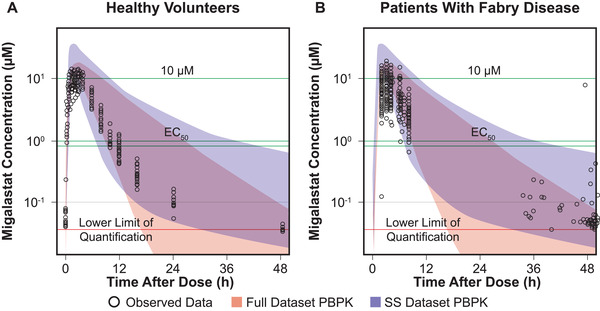
Observed and predicted plasma migalastat concentration‐time profiles in healthy human subjects and patients with Fabry disease for the full dataset physiologically based pharmacokinetic (PBPK) and steady‐state PBPK models following oral administration of migalastat 123 mg every other day. EC_50_, half‐maximal effective concentration; h, hour; SS, steady‐state. Colored strips indicate 95% prediction interval; dark purple indicates overlapping prediction interval. The full dataset PBPK and steady‐state dataset PBPK models were extrapolated to humans by fitting plasma concentration‐time profiles from phase 1 (healthy volunteers, AT‐1001‐015^16^) and phase 3 (patients with FD, AT1001‐011^19^) at the oral dose of 123 mg every other day.

The Full Dataset PBPK and Steady‐state Dataset PBPK models were extrapolated to humans by fitting plasma concentrations in healthy volunteers and patients with FD after a single oral dose of 123 mg. Given that the goodness of fit was adequate for plasma, with the model developed in mice (with human clearance parameters) describing the concentration‐time profiles in humans (Figure 5), tissue concentrations were then predicted from each model. The predicted human tissue concentrations were compared with the half‐maximal effective concentration (EC_50_), which represented the concentration corresponding to 50% migalastat binding to migalastat‐amenable α‐Gal A enzymes in vitro.

**Figure 6:**
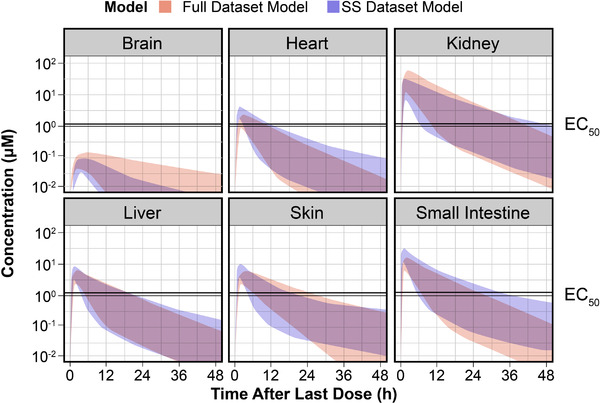
Predicted human tissue migalastat concentration‐time profiles at steady state following oral administration of migalastat 123 mg every other day using the full dataset physiologically based pharmacokinetic (PBPK) model and steady‐state dataset PBPK model. h, hour; SS, steady‐state. Color strips indicates 95% prediction interval; dark purple indicates overlapping prediction interval, and steady‐state dataset PBPK model. The full dataset and steady‐state dataset PBPK models are shown as 95% prediction interval. Plasma concentration‐time profiles for migalastat were predicted in human tissues using the full dataset and steady‐state PBPK models at the oral dose of 123 mg every other day.

Both the Full Dataset PBPK Model and the Steady‐state Dataset PBPK Model predicted similar times above the EC_50_ in a single dosing interval at steady state. Following steady‐state dosing of migalastat 123 mg every other day in humans, predicted human migalastat concentrations were highest in the kidney, followed by small intestine, skin, liver, heart, and brain (Figure 6). At steady‐state dosing with the labeled dose of 123 mg every other day, predicted human migalastat concentrations were expected to exceed the EC_50_ (0.82‐1 μM; unpublished observations) in all tissues except brain for ≥95% of all subjects.

### PBPK Model Fit of Agalsidase Beta Compared With Observed Data in Mice

A physiologic model of agalsidase beta is expected to consist of tissue macrophage uptake from plasma into intracellular volumes of the liver and spleen and M6PR‐mediated drug internalization (or target‐mediated drug disposition [TMDD]) for other tissues. Macrophage uptake was not implemented in the current version of PK‐Sim or MoBi, so various models were attempted. Modeling of agalsidase beta concentrations was initially attempted with a simple active transporter uptake model, which was able to describe the concentrations in all tissues except the liver and required high metabolic clearance in plasma that is inconsistent with nonspecific peptide hydrolysis.

An agalsidase beta PBPK model (Table 2) was reasonably fitted to the mouse data by assuming that all tissues with detectable levels (including the liver and spleen) took up agalsidase beta via M6PR TMDD (95% prediction intervals; Figure 3). A larger incongruity was observed between predicted and observed concentrations at 24 hours, when the tissue concentrations were overpredicted and the plasma concentration was underpredicted. A physiologic rationale for the dip in tissue exposure at 24 hours compared with the trajectory at other times is lacking. Given that the tissue distribution data were contributed by only a few mice at each timepoint, the variation may be because of variability. The choice was made to fit the model to the point at 48 hours, which better agreed with the other timepoints and predicted higher tissue exposure. Plasma concentrations were only assessed up to 24 hours by study design, so it was more difficult to assess whether this timepoint represents an aberration. The persistent plasma concentration at 24 hours may be because of endogenous agalsidase interference, as lysosomal enzymes have the capacity to escape trafficking by M6PR and become secreted.^32^ However, the misfit in plasma concentration at 24 hours has little influence on the overall model, as the majority of the drug has been distributed and accounted for, and the level of agalsidase at 24 hours is negligible compared with peak plasma concentration. Therefore, accumulation is not expected to be of concern with an every‐other‐week dosing regimen.

**Table 2:** Relevant Parameters for the Agalsidase Beta PBPK Model

Parameter	Optimized Value	Source/Rationale
Is a small molecule	No	Agalsidase beta is a recombinant enzyme made of 2 subunits of 398 amino acids
Molecular weight, kDa	102.4	Agalsidase beta is a homodimeric glycoprotein, with each monomer having a molecular weight of 51.2 kD^11^
Peptide hydrolysis–intracellular/interstitial specific clearance, (min^‐1^)	2 × 10^−4^	Fitted
Peptide hydrolysis–Intracellular/interstitial enzyme concentrations	1 μM	Assumed to be located in all organs (both intracellular and interstitial compartments) and plasma equally
M6PR K_d_, nM	3.0	Dwyer et al, 2020^41^
M6PR K_off_, (sec^‐1^)	1.0	Fitted
M6PR K_internalization_, (min^‐1^)	0.05	Fitted
M6PR maximum enzyme concentrations	0.42 μM	Fitted
Relative expression of M6PR in liver	100%	Fitted
Relative expression of M6PR in spleen	42%	Fitted
Relative expression of M6PR in kidney	6%	Fitted
Relative expression of M6PR in heart	2%	Fitted

K_d_, equilibrium dissociation constant; K_off_, off‐rate constant; min, minute; M6PR, mannose‐6‐phosphate receptor; PBPK, physiologically based pharmacokinetic.

Compared with the active transport process, the receptor (M6PR) has high affinity for the ligand (agalsidase beta) and becomes internalized along with the ligand. The modeling process revealed that agalsidase beta did not easily diffuse across cell surfaces and that the rapid decline in plasma was mainly driven by rapid tissue sequestration into the liver and spleen, rather than rapid plasma hydrolysis. Because most of the infused dose was nearly accounted for before the addition of the plasma hydrolysis process, little agalsidase beta was expected to be available for other unstudied tissues and organs, and agalsidase beta levels in other tissues and organs were expected to be negligible.

### Predicted Agalsidase Beta Tissue Concentrations in Humans Using Mouse‐to‐Human Extrapolation

The best agalsidase beta PBPK model in mice was attempted for the mouse‐to‐human extrapolation. However, this extrapolation was unsuccessful, as the predicted human plasma concentrations exceeded observed human plasma concentrations by ≈20‐fold. Several changes to the mouse PBPK were attempted, including modeling liver and splenic uptake as direct passive diffusion, but all were unsuccessful at removing the large difference between predicted and observed plasma concentrations in humans. In particular, the rapid drug disposition to the liver and spleen compartments in mice cannot be replicated by optimizing M6PR kinetics and expression in humans. These 2 organs sequestered most of agalsidase in animal and human studies and primarily uptake lysosomal enzymes via macrophage mannose receptors on the plasma membrane, which was not successfully modeled in mice. The importance of liver and splenic uptake at driving agalsidase plasma concentrations was tested by removing the processes in the mouse model, which then produced grossly overpredicted plasma concentrations and a biodistribution pattern similar to the human model prediction, suggesting that the high predicted human plasma concentrations resulted from insufficient liver and splenic uptake. Therefore, agalsidase beta tissue concentrations were only modeled in mice and not extrapolated to humans.

## Discussion

The present study reported PBPK models describing plasma and tissue concentration‐time data after oral administration of migalastat or intravenous administration of agalsidase beta in mice. The PBPK model of migalastat in mice could be extrapolated to humans based on good agreement between observed and predicted human plasma concentration‐time data, but the PBPK model for agalsidase beta in mice could not be extrapolated to humans because of poor agreement (>20‐fold) between predicted and observed human plasma concentration‐time data. A comparison of biodistribution of the 2 compounds based on the PBPK models in mice is discussed, followed by predicted migalastat concentration‐time data in humans and theoretical descriptions of expected human biodistribution of agalsidase beta.

The PBPK model for migalastat in mice showed that migalastat was broadly distributed across many FD‐relevant tissues and eliminated more slowly in tissue than in plasma, which suggested the presence of the same reversible tissue‐binding mechanism across tissues (with the exception of the spleen, which had higher tissue retention). This is consistent with lysosomal sequestration given that lysosomes are similar and present across different cell types and easily lysed in assay conditions to release free migalastat for overall concentration determination.

The PBPK model of agalsidase beta assumed TMDD via M6PRs and was able to describe the tissue and plasma concentration‐time profile in mice. Agalsidase beta concentrations were highest in the liver and spleen, 2 organs that are not known to be involved in FD pathology and absent in the small intestine and brain. Sequestration of agalsidase beta in the liver and spleen was consistent with biodistribution studies of other lysosomal enzymes^33^, ^34^; it has been hypothesized that the rapid clearance of the infused enzymes to liver and spleen limits the distribution to the tissues of interest for numerous lysosomal ERTs.^35^ Based on existing knowledge about therapeutic proteins, agalsidase beta is unable to freely distribute across cell membranes without the assistance of receptor‐mediated endocytosis, which also directs the trafficking of exogenous α‐Gal A to lysosomes. In mice, the decline of plasma α‐Gal A concentration was almost completely accounted for by drug distribution into liver, spleen, kidney, and heart, with residual clearance explained by a minor peptide hydrolysis process, suggesting that agalsidase beta was not appreciably distributed into other tissues. This inference was consistent with the results of a previous mouse study using agalsidase alfa, which showed that agalsidase alfa was only detected in the liver, spleen, kidney, heart, adrenal gland, testis, and bone marrow, some of which only had detectable levels in vascular endothelial cells and was not detected in the 34 other organs and tissues assessed.^7^

Although the concentrations of migalastat and agalsidase beta could not be directly compared, migalastat was distributed to a greater extent in kidney and intestinal tissues, whereas only a small percentage of the infused agalsidase beta dose was distributed into the kidneys, and no agalsidase activity was detected in the small intestines in mice. Based on our findings, migalastat readily diffused into most cells and tissues, whereas agalsidase beta was only taken up in a subset of organs. Taken together, these results suggest that migalastat had broad tissue distribution in Fabry‐related tissues. One limitation of the PBPK models was that migalastat and agalsidase beta tissue concentration‐time profiles were assessed in wild‐type mice. The PBPK approach requires comprehensive and validated modeling of the anatomy and physiology of the organism, but the knowledge of FD‐related changes is insufficient. Therefore, the PBPK models were built assuming wild‐type mice, and wild‐type mice were used as the observation. For agalsidase beta, biodistributions were shown to be comparable between wild‐type and hR301Q α‐Gal A Tg‐KO mice 2 hours after dose, indicating that the results from the wild‐type mouse study are predictive of the biodistribution of agalsidase beta in FD mice. For migalastat, the mutant mice had lower 2‐hour concentrations in kidneys, liver, and small intestine, but the ranges of exposure overlapped significantly. Because the kidneys and small intestines were 2 organs with the highest migalastat concentrations, a 3‐fold reduction in these tissues is still expected to result in significant exposure.

The PBPK models for migalastat reliably predicted migalastat concentrations in mouse tissues, and the extrapolated concentrations in human plasma were consistent with the observed plasma concentrations in healthy human subjects and patients with FD,^16^, ^26^ supporting its utility in predicting human tissue concentrations. In clinical studies with migalastat, the peak plasma concentration of 10 μM was associated with stabilized renal function and improvements in gastrointestinal symptoms.^19^, ^21^, ^36^ Our PBPK extrapolation to humans showed that at steady‐state dosing of 123 mg every other day, the clinically effective dose associated with the peak plasma concentration of 10 μM, heart, kidney, liver, skin, and small intestine in almost all subjects were predicted to attain the EC_50_. The prediction of adequate tissue concentrations in Fabry‐related tissues is consistent with efficacy data from phase 3 studies. Migalastat stabilized renal function (consistent with the drug distributing into the kidney) and significantly decreased LVMi (consistent with the drug distributing in the heart) in ERT‐experienced patients in the 18‐month phase 3 ATTRACT study^21^ and in the 24‐month phase 3 FACETS study.^19^ Similarly, migalastat treatment also improved gastrointestinal symptoms in ERT‐naive patients over a 24‐month phase 3 FACETS study.^19^

Extrapolation of the agalsidase beta model to humans could not be interpreted because there was a 20‐fold overprediction of plasma concentrations in humans. Unfortunately, compared with PBPK modeling of small molecules, PBPK modeling of therapeutic proteins is still in its infancy,^37^ and the failure to extrapolate mouse data to human predictions for agalsidase beta was, at least in part, a limitation in the ability to account for all aspects of drug‐organism interactions with the current software. The failed extrapolation implied either that the proposed PBPK model did not capture the actual physiology of agalsidase beta disposition or that physiological differences existed between mice and humans that made the extrapolation impossible. In particular, the failure to accurately predict human plasma concentration‐time data may have been related to the inability to model the direct plasma‐to‐intracellular uptake process by hepatic Kupffer cells and splenic macrophages, an important process that drives the plasma pharmacokinetics of agalsidase beta and other lysosomal ERTs.^5^ This process was not available within the software, and attempted workarounds were unsuccessful.

Although the mouse‐to‐human extrapolation of the agalsidase beta PBPK model was unsuccessful, the biodistribution of agalsidase beta in humans can be qualitatively evaluated based on its property as a lysosomal enzyme and its clinical efficacy. The findings of high liver and splenic concentrations in the mouse study presented in this article were consistent with the mouse biodistribution studies of agalsidase alfa^11^ and other lysosomal enzymes,^33^, ^34^ suggesting that agalsidase beta biodistribution is typical of lysosomal enzymes. Liver and spleen directly scavenge lysosomal enzymes from the plasma, a more efficient process than passive diffusion from plasma to the tissue interstitial space followed by tissue uptake at the interstitial membrane. Humans also possess the same mechanisms for mannose receptor uptake,^38^ so high liver and splenic concentrations are to be anticipated. Clinically, agalsidase beta therapy was associated with near‐complete clearance of glycolipid in the vascular endothelial cells of the liver, heart, and skin, a subset of kidney cell types, and cardiac endocardium.^8^, ^9^, ^39^, ^40^ However, postmortem assessment of a patient who died after 2.5 years of agalsidase beta therapy suggested limited tissue penetration, with glycolipid deposits observed in the heart myocytes and fibroblasts as well as kidney glomerular epithelium and tubular epithelial cells.^40^ Therefore, agalsidase beta may have a limited distribution to heart and some cells of the kidney.

In conclusion, migalastat distributes freely into various FD‐relevant tissues in mice including the heart, kidney, and small intestine, and its retention in these tissues is consistent with lysosomal sequestration. Agalsidase beta concentrations in mice are highest in the liver and spleen, 2 organs that are not known to be involved in FD pathology, lower in the heart and kidney, and absent in the small intestine and brain. The extrapolated migalastat human PBPK model linked the observation of apparent clinical efficacy in the heart, kidney, and the gastrointestinal tract associated with peak plasma concentration of 10 μM to predictions of in vitro EC_50_ attainment in the various target organs of interest. Although the extrapolation of the agalsidase beta PBPK model was unsuccessful, mice data suggested that rapid sequestration of the lysosomal enzyme in the liver and spleen is likely an important driver of its plasma pharmacokinetics. Taken together, our result show that migalastat may confer protection for important organs in the treatment of FD.

## Conflicts of Interest

Y.S.W. is an employee of and V.S. is a subcontractor for Nuventra Pharma Sciences, which was contracted by Amicus Therapeutics, Inc. for analysis of this study. R.K., J.‐S.S., A.G., L.D., and R.H. are former employees of and hold stock in Amicus Therapeutics, Inc. Y.L., A.P., L.M., P.‐C.T., and F.K.J. are employees of and hold stock in Amicus Therapeutics, Inc. A.M.D. has nothing to disclose. R.S. has served as a consultant and received honoraria from 4D Molecular Therapeutics, Amicus Therapeutics, Inc., and Prevail Therapeutics.

## Funding

This study is supported by Amicus Therapeutics, Inc.

## Author Contribution

Y.S.W., V.S., R.K., F.K.J. contributed to study design. R.K., Y.L., J.S., A.G., L.D., A.P., L.M., P.T., R.H. and F.K.J. participated in data collection. Y.S.W., R.K., V.S., J.S., A.M.D. and F.K.J. participated in data analysis and data interpretation. Y.S.W., K.R., F.K.J. and A.M.D. drafted the manuscript. All authors critically reviewed the manuscript and approved the manuscript for publication.

## Supporting information

Supplementary information

## Data Availability

All relevant data are reported in this manuscript or provided in accompanying supplemental materials.
