# Supplementary material for: Migalastat Tissue Distribution: Extrapolation From Mice to Humans Using Pharmacokinetic Modeling and Comparison With Agalsidase Beta Tissue Distribution in Mice
Source: Clin Pharmacol Drug Dev. 2021 Apr 19;10(9):1075–88. doi: 10.1002/cpdd.941 (PMC8453552; doi:10.1002/cpdd.941)
Supplement: Supplementary file 1 — Supplementary information [file CPDD-10-1075-s001.docx]

**SUPPLEMENTARY INFORMATION**

**Supplemental Figure 1.** Migalastat Tissue Concentration at 2 Hours Postdose in Wild-type Mice and a Mouse Model of Fabry Disease^a,b^


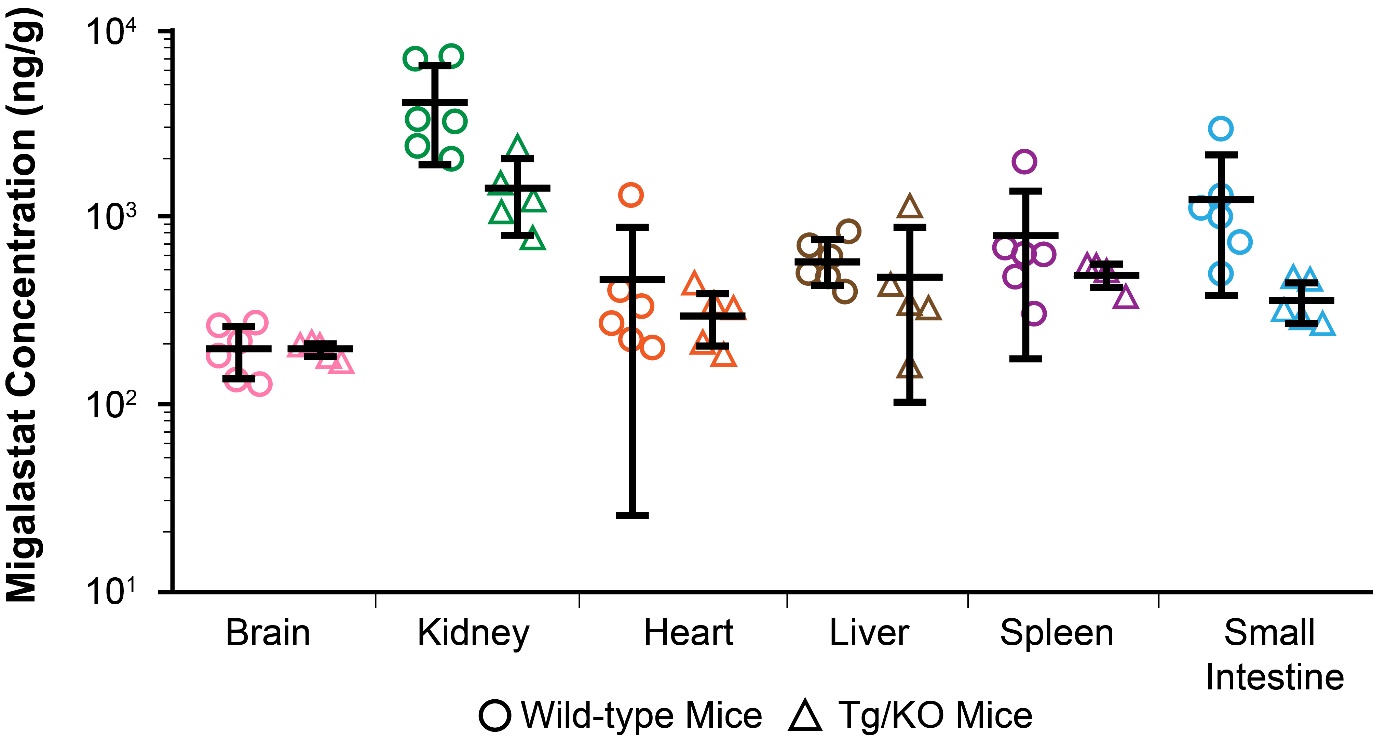


h, hour; KO, knockout; SD, standard deviation; Tg, transgenic.

^a^The hR301Q α-Gal A Tg/KO mouse model of Fabry disease was assessed.

^b^Mean (SD) are plotted.

Migalastat concentrations were compared 2 hours postdose in tissues of wild-type and hR301Q α-Gal A Tg/KO mice (n=5) receiving oral doses of migalastat (30 mg/kg 3 times per week for 12 doses).

**Supplemental Figure 2.** Agalsidase Beta Tissue Concentrations at 2 Hours Postdose in Wild-type Mice and a Mouse Model of Fabry Disease^a^


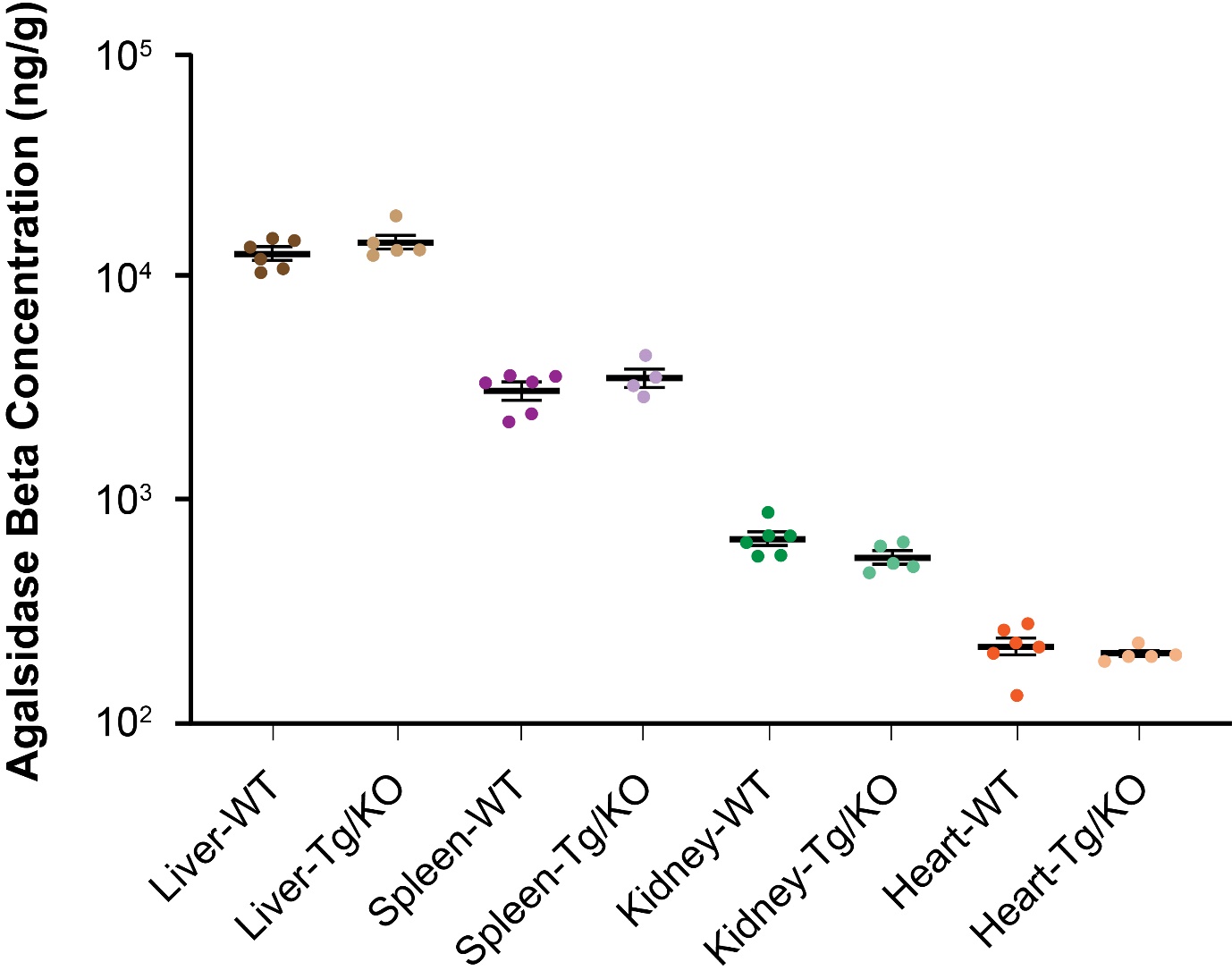


h, hour, KO, knockout; SEM, standard error measurement; Tg, transgenic; WT, wild-type.

^a^Error bars represent mean ± SEM.

At 2 hours postdose, agalsidase beta concentrations were compared in tissues of wild-type and hR301Q α-Gal A Tg/KO mice (n=5) receiving intravenous dosing with agalsidase beta (1 mg/kg biweekly for 2 doses). Endogenous α-Gal A concentration in wild-type mice was subtracted from the values.

**Supplemental Figure 3.** Migalastat Plasma Concentrations at Each Dose Level

**
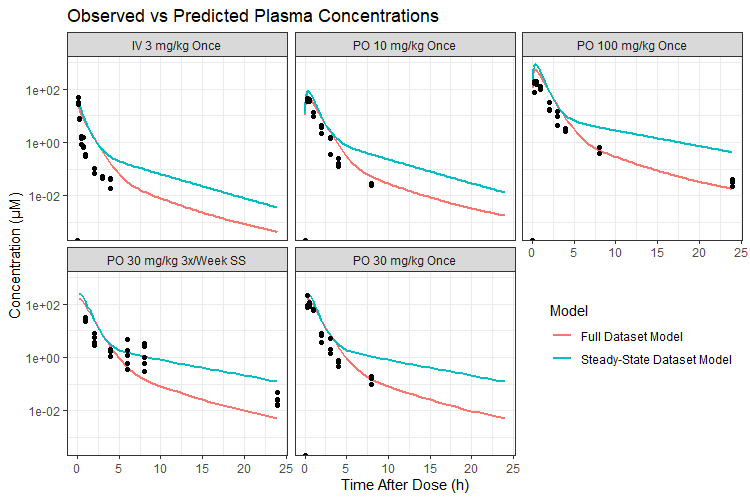
**

h, hour; IV, intravenous; PO, oral administration; SS, steady-state.

**Supplemental Figure 4.** Observed and PBPK Model-Predicted Migalastat Tissue and Plasma Concentration-Time Profiles in Mice Following a Single Oral Dose of 100 mg/kg Migalastat


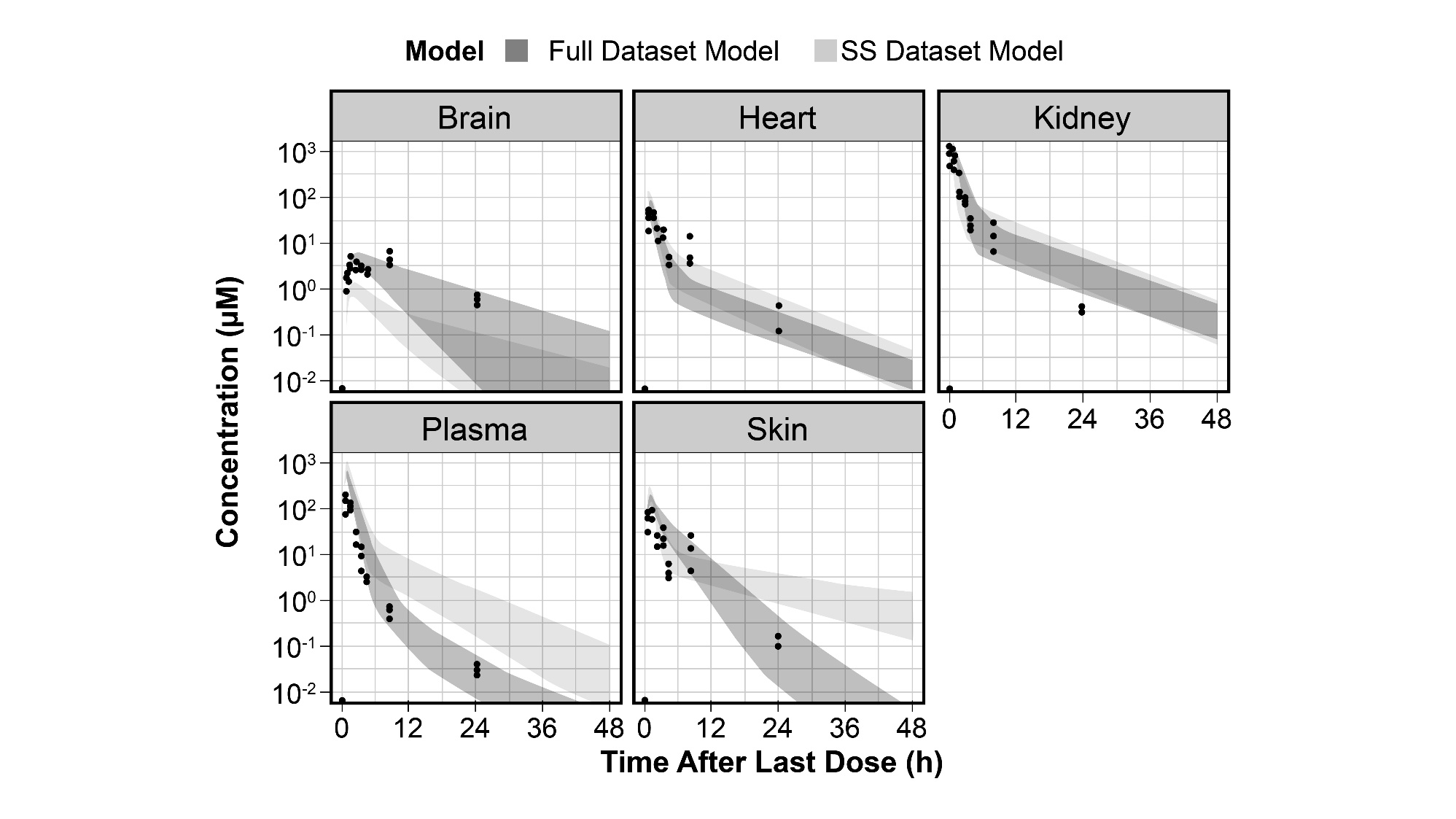


h, hour; PBPK, physiologically based pharmacokinetic; SS, steady state.

Dots are observed values; colored strips are predicted values (95% prediction interval); darkest gray indicates overlapping prediction interval.

A mouse PBPK model was developed and fitted to observed mouse migalastat concentrations following a single oral dose (100 mg/kg).

**Supplemental Figure 5.** Migalastat Tissue Concentrations in Wild-type Mice Following Single and Steady-State Dosing Comprising Free and Lysosomal Bound Migalastat


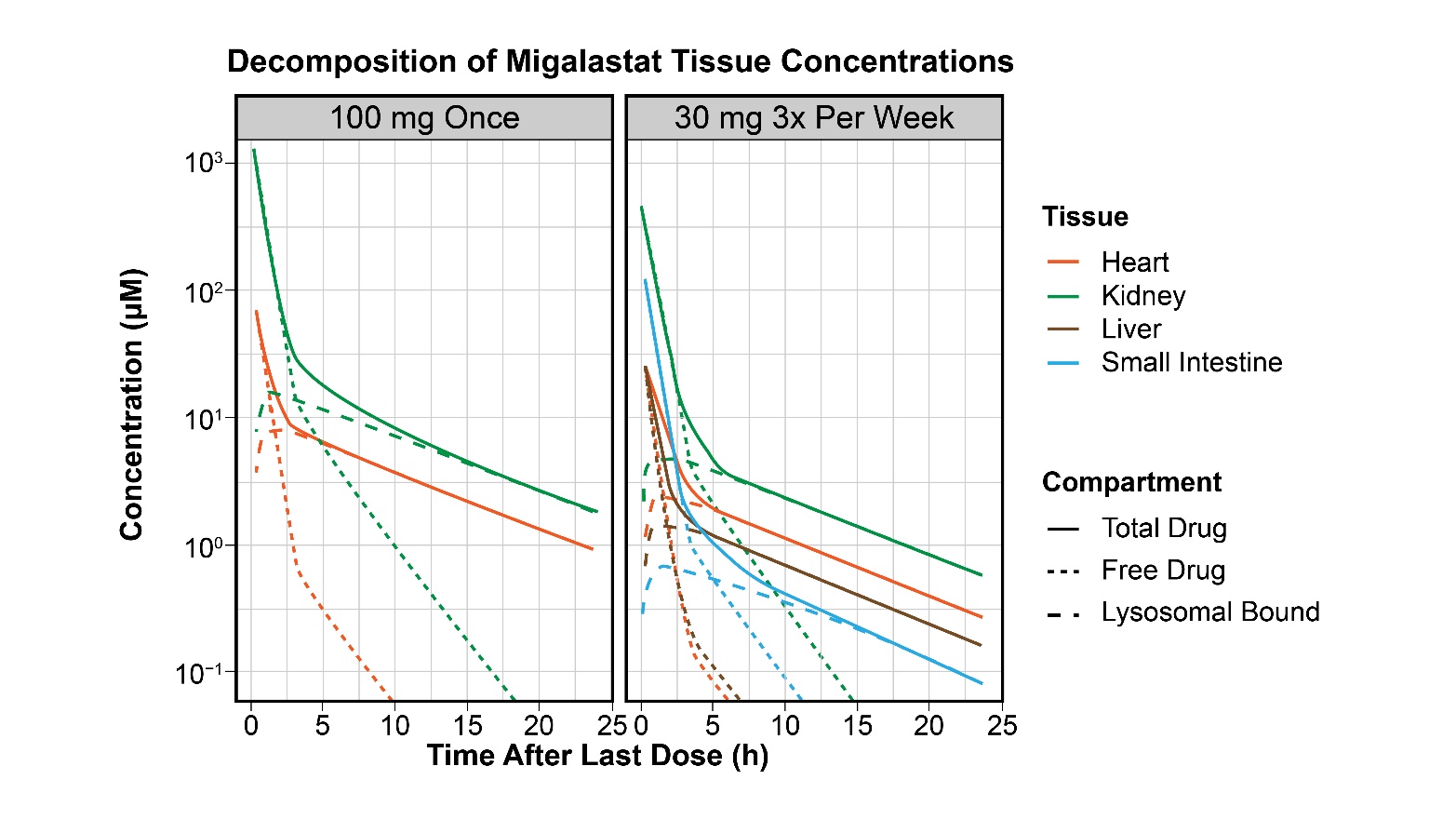


h, hour; PBPK, physiologically based pharmacokinetic model.

This exploratory analysis was performed to assess whether this approach was appropriate for the PBPK model.

The initial tissue concentrations were assumed to primarily comprise free (unbound) migalastat, which freely diffuses across membranes and falls in parallel with plasma migalastat; the expected free tissue concentrations were subtracted from the overall tissue concentrations to impute the lysosomal-bound concentrations. The kinetics for lysosomal uptake and release were modeled based on the tissue lysosomal-bound concentrations.
